# A Case of Gouty Heart, with Notes of Autopsy

**Published:** 1882-01

**Authors:** H. R. Hopkins


### A Case of Gouty Heart, with Notes of Autopsy.

Reported by H. R. Hopkins, M. D.

C. T. C., æt. 62, was plainly of gouty ancestry. Gout having frequently visited his father and paternal aunts, and being a constant companion of his brother, while a sister had suffered for years with a chronic eczema of a supposed rheumatic character.

He had seen the prosperous and comfortable side of life; was a successful banker; had been a man of correct habits; was probably a large eater, and for years accustomed to take with his meals large quantities of ice water, four to six goblets.

In early manhood he suffered severely from gall-stone colic, but for the last twenty years had enjoyed average health, with only an occasional bilious attack. He was very fond of the saddle, and in summer and winter could be seen almost any day upon the road.

Early in January of this year, 1881, he had a severe attack of suffocative pain in the region of the heart, accompanied with urgent dyspnœa. The attack came without warning and could not be traced to exposure or strain. The pulse was irregularly intermittent, the heart's sounds confused and its action wobbling the area of cardiac dullness was thought to be rather increased towards the right. The surface and extremities were cool, the mind clear and very anxious and apprehensive. This was the beginning of an illness which lasted nearly three months and furnished continuous signs of heart failure with some quite serious and complicating sequela. The pulse was rapid and intermittent throughout, and as soon as the heart's action became sufficiently quiet to allow accurate observation, a reduplication of the first sound was heard, which continued until thoroughly convalescent. The sounds would be represented by lub-ud-tip, but I quite often for days would hear the fourth sound like this lub-ud-te-tip. But the first was the more frequently heard. About the second week serious œdema of the lungs took place, and for nearly a month he was obliged to spend his time in a chair. During most of his illness marked dropsy of the lower limbs was also present. All of these conditions slowly improved, and he went about his work for a few of the summer months and was occasionally in the saddle. In the early autumn he again failed, showing many of the same signs as in his winter attack; walking became well nigh impossible with bad digestion and worse nights. In desperation he spent six weeks in New York under the most favorable auspices and the most eminent medical advice, but failed gradually and surely.

On the morning of the 11th inst., after passing a fair night and eating a light and well-relished breakfast, lay gently back upon the pillow, and with scarcely a moment's warning expired.

The autopsy was performed 48 hours after death, in the presence of Drs. Burwell, Folwell and Cary. The right pleural cavity contained three pints of fluid, but showed no other signs of pleurisy. The left pleural cavity was normal throughout, save slight adhesions between the pleural surface in the interlobar fissures. The lungs were normal. The pericardium contained 13 drams of clear fluid, but no signs of inflammation. The heart was large, weighed twenty-three ounces, and the aortic valves held water. The endocardial surface was mottled with light colored spots.

In the cavity of the left ventricle was an old thrombus, firm to the touch, gray and laminated, oval in shape, larger than a silver dollar, and at its center a sixth of an inch in thickness. This was firmly adherent throughout, and when detached left an irregular spot of from one-half to two-thirds of an inch in diameter, denuded of endocardial membranes.

The entire wall of this ventricle, in the locality, showed typical signs of fibroid degeneration. The valves were normal in shape, but all showed the results of inflammation. Microscopic examination demonstrated marked fatty degeneration.

The liver weighed five pounds, the kidneys nine ounces each, and were all granular and over firm to cut and feel.

This case seems worth recording as a typical instance of the remarkable fatality among our active men of 60 to 65 years of age. Among the prominent families of our city we see a community of widows, the large majority of whose husbands have died between the years just mentioned.

Again, the case singularly illustrated the persistence of certain diseased tendencies. The subject was a man of remarkable judgment and unusual self-discipline. He was perfectly aware of his peculiar inheritance, and in general his life was ordered with the hope of keeping the evil tendencies of that inheritance in check. His occupation and tastes were favorable to the promotion of health. He had been remarkably preserved from the depressing effects of poverty, care, reverses, or disappointments. He had been loved and honored at home and abroad, and yet he was cut off in the zenith of his old age, with the visceral manifestation of his family's enemy, "inherited gout."

On looking over his life and habits, there was nothing I could find to criticize, save the fact that he was over fond of sweets, pies, preserves and candy, and his use of them amounted to intemperance. Then he was for years quite intemperate in the use of ice water, but we would hardly make of this a serious dietetic error.

The condition of the kidneys was that of chronic Bright's disease, and yet up to the last week of his life his urine contained no albumen. This fact is worthy of note in connection with the importance attached to the presence or with the absence of albumen from the urine. Plainly, the absence of albumen gives no warrant for the opinion that the kidneys are healthy; and it seems to be equally plain that in the presence of inherited gout there are no such things as "trifling dietetic errors."

